# Case Report: Hepatic sarcoidosis-like reaction from neoadjuvant pembrolizumab in early-stage triple-negative breast cancer

**DOI:** 10.3389/fimmu.2025.1589191

**Published:** 2025-06-18

**Authors:** Yujing Tan, Yu Wang, Xiaoyan Liu, Qin Ma, Cheng Zeng, Aihua Zhu, Xiaoying Sun, Fei Ma, Jiani Wang

**Affiliations:** ^1^ Department of Medical Oncology, National Cancer Center/National Clinical Research Center for Cancer/Cancer Hospital, Chinese Academy of Medical Sciences and Peking Union Medical College, Beijing, China; ^2^ Breast Radiotherapy Ward, Department of Radiotherapy, Shanxi Province Cancer Hospital, Shanxi Hospital Affiliated to Cancer Hospital, Chinese Academy of Medical Sciences, Cancer Hospital Affiliated to Shanxi Medical, Shanxi, China; ^3^ Department of Respiratory and Critical Care Medicine, Peking Union Medical College Hospital, Chinese Academy of Medical Sciences & Peking Union Medical College, Beijing, China; ^4^ Department of Medical Oncology, Cancer Hospital of HuanXing ChaoYang District, Beijing, China

**Keywords:** triple-negative breast cancer, pembrolizumab, sarcoidosis-like reaction, neoadjuvant immunotherapy, early breast cancer

## Abstract

Drug-induced sarcoidosis-like reaction (DISR) is a rare adverse event associated with immunotherapy. Currently, there is no standardized treatment protocol for DISR linked to immune checkpoint inhibitors (ICIs). This study presents a case of an early-stage triple-negative breast cancer (TNBC) patient who developed hepatic sarcoidosis-like reactions during neoadjuvant pembrolizumab therapy. We provide an overview of ICI-induced sarcoidosis-like reactions in cancer patients, including incidence, mechanisms, clinical manifestations, treatment, and prognosis. Additionally, we discuss the significance of one-year adjuvant immunotherapy for early-stage TNBC patients who achieved pathological complete response after neoadjuvant therapy, offering insights for individualized therapeutic strategies in this population.

## 1 Case presentation

### 1.1 Treatment for locally advanced triple-negative breast cancer

A female patient in her 40s, premenopausal, was diagnosed with locally advanced TNBC. Pathological analysis confirmed invasive ductal carcinoma (IDC), grade III, negative status of estrogen receptor (ER), progesterone receptor (PR) and HER2, a high Ki-67 index of 80%, and a combined positive score of 10% for programmed death ligand 1 (PD-L1) expression. Biopsies of the left supraclavicular and axillary lymph nodes showed cancer cells. MRI and whole-body PET-CT scans indicated a clinical stage of T2 (2.4cm x 1.7cm) N3c (positive left supraclavicular lymph nodes) M0.

Inspired by the promising outcomes from the KEYNOTE-522 study (1, 2), the patient would receive four cycles of neoadjuvant paclitaxel (175 mg/m²) plus pembrolizumab (200 mg) every three weeks, followed by four cycles of epirubicin (90 mg/m²) and cyclophosphamide (600 mg/m²) (EC regimen) + pembrolizumab. However, a contrast-enhanced MRI after six cycles revealed a suspicious lesion (2.4 x 2.4 x 3.0 cm) with cystic changes in the left lobe of the liver (**Supplementary Figure S2A**). To mitigate potential immune checkpoint inhibitors (ICIs)-related adverse events (AEs) and enhance the efficacy of neoadjuvant therapy, cycle 7 was treated with EC chemotherapy alone, omitting ICIs. After seven cycles, the tumor lesions were confirmed as clinical CR. The efficacy of neoadjuvant therapy is summarized in **Table 1**; **Supplementary Figure S3**.

**Table 1:** Efficacy of neoadjuvant therapy of the early-stage TNBC patient.

Target lesions	Baseline (cm)	2 Cycles (cm)	4 Cycles (cm)	7 Cycles or before surgery (cm)
Left breast	2.4 x 1.7	1.3 x 1.2	0.5 x 0.3	Unmeasurable
Left axillary lymph node	1.4	0.7	0.7	0.5

The patient underwent modified radical surgery after neoadjuvant therapy. Postoperative pathology revealed a pathological complete response (pCR). Due to the uncertain nature of the liver lesion, adjuvant pembrolizumab was postponed. Adjuvant radiotherapy was administered according to the radiation therapists.

### 1.2 ICI-induced hepatic sarcoidosis-like reaction and follow-up

**Figure 1:**
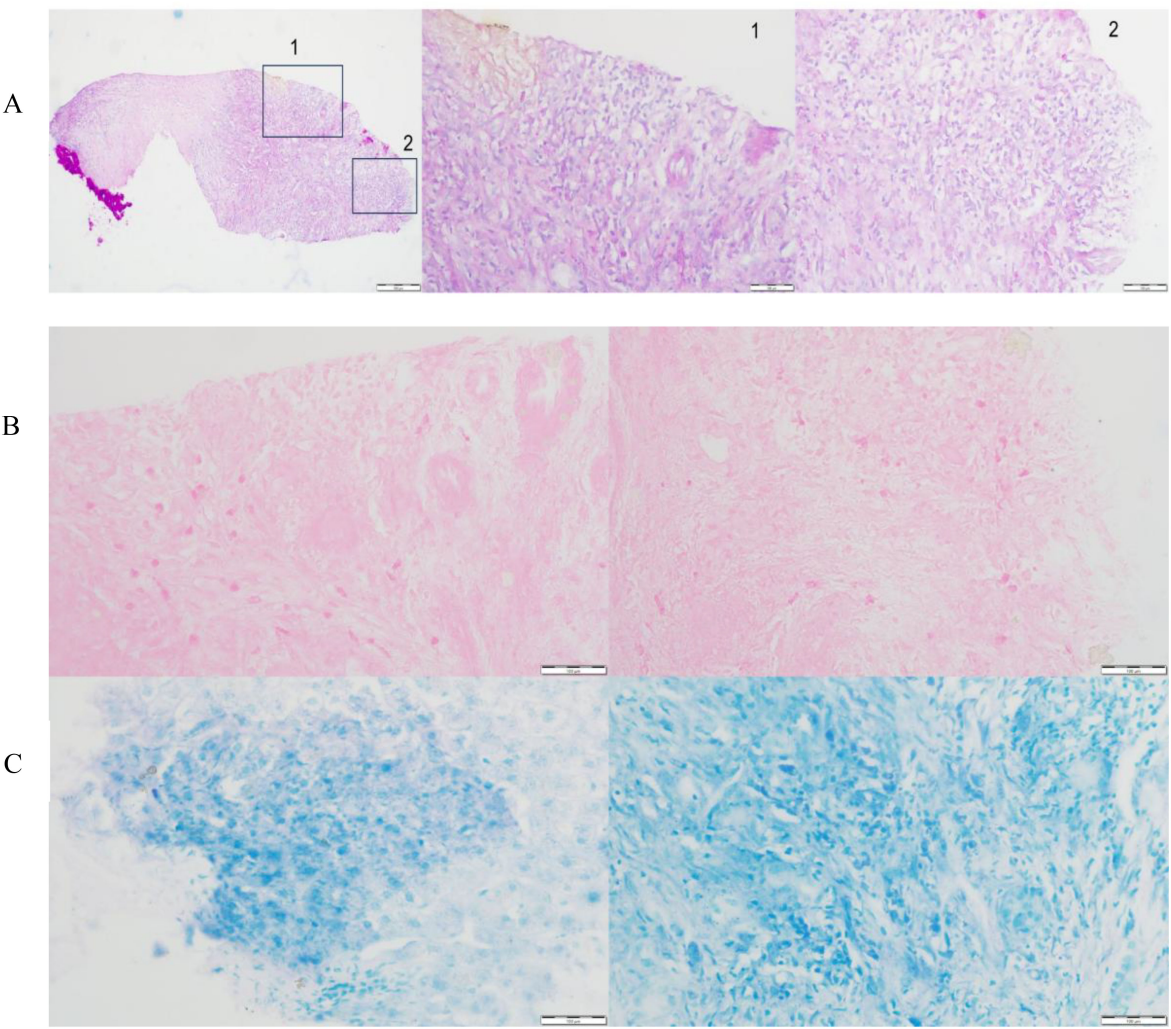
Pathological evaluation associated with tuberculosis of the liver tissue. **(A)** High levels of inflammatory cells and hyperplasia fibrotic cells were observed in the marked areas, indicating chronic granulomatous inflammation. Special staining of antacid-TB **(B)** and PAS **(C)** were negative.

A liver puncture biopsy unraveled some swelling hepatocytes accompanied by local steatosis and siltation, as well as local epithelioid cell nodules with necrosis, which are infiltrated by high levels of inflammatory cells and hyperplasia fibrotic cells (**Figure 1A**). Immunohistochemistry: CD34 (vascular +), CD163 (partial +), CD68 (partial +), Ki-67 (10% +), GPC-3 (-). Special staining: PAS (-) (**Figure 1B**, 40X), weak antacid stain (-), silver hexamine (-), antacid-TB (-) (**Figure 1C**, 40X). Peripheral blood and hepatic cystic fluid were further tested for multiple nucleic acid high-throughput sequences (covering 44 bacteria, 13 fungi, 7 DNA viruses, 4 parasites, and 7 atypical pathogens), as well as inflammatory markers (including CRP, PCT and ESR), various cytokines, T-cell subsets, G and GM tests, to identify potential pathogens. As a result, no evidence of infection in a spectrum of pathogens was observed. Additionally, the patient denied any infection-related symptoms during the overall course of disease, such as fever, cough, sputum, and diarrhea, *etc.* Doctors draw out the opinion that liver metastasis and infection are not considered, while tuberculosis needs to be precluded.

**Figure 2:**
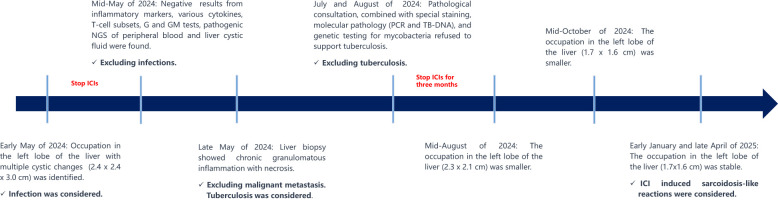
Overview of the diagnosis and treatment of the liver lesion in the left lobe.

The patient then consulted two specialized hospitals and came up with similar conclusions. Primary T/B-spot screening was negative. Further examination associated with tuberculosis using the biopsy, including pathology consultation, molecular pathology (such as PCR, TB-DNA), special staining, and genetic testing for mycobacteria, unraveled negative results. The pathology indicated chronic granulomatous inflammation with necrosis. Several months after discontinuing immunotherapy, follow-up MRI showed a reduction in the liver lesion’s size (Mid-August of 2024: 2.3 x 2.1 cm, Mid-October of 2024 to late April of 2025: 1.7x1.6 cm), confirming its stable condition (**Supplementary Figures S2B-S2D**). **Figure 2** summarizes the diagnosis and treatment process for the liver lesion.

Since the patient reported no liver discomfort or functional abnormalities, combined with the reduction of the sarcoidosis-like lesion after discontinuing ICIs, hormonal or anti-tuberculosis therapy was deemed unnecessary. The enlarged liver lesion exhibited no anomalies and spontaneously diminished without medical intervention. Therefore, the hepatic cystic lesion that arose during pembrolizumab treatment is likely an ICI-induced AE.

## 2 Discussion

### 2.1 ICI induced sarcoidosis-like reaction

ICI-induced sarcoidosis-like reaction (ISR) is relatively rare, which can be attributed to anti-PD-1 therapy, anti-PD-L1 therapy, and anti-CTLA-4 therapy (3). In a retrospective real-world study, the incidence of DISR caused by anti-PD-1 treatment, anti-CTLA-4 treatment, and anti-PD-1 + anti-CTLA-4 treatment was 3.7% (11/297), 3.7% (3/81), and 6.3% (5/79), respectively (4). In another study, the incidence was reported as 2.4% (2/83) (5). The median interval from initiation of ICIs to DISR has been documented as 5.5 months (range: 2.3-13.5 months) (6, 7), which is highly consistent with the present case. The mechanism of ISR remains unclear. Possibly, the use of ICIs triggers a series of enhanced immune responses in a pathologic way, leading to systemic sarcoidosis-like reactions (4, 8).

The clinical manifestations of ISR vary, which mainly present with enlarged lymph nodes. It also exhibits sarcoidosis-like reactions in distinct organs, accompanied by fever, dysfunction of the liver or kidney, or endocrine disorders (**Supplementary Table S1** **) (** 5, 9–12). Nevertheless, hepatic sarcoidosis-like reactions from neoadjuvant pembrolizumab have not been reported.

Most patients with ISR are asymptomatic, and do not require systemic therapy (8). In general, ISR can automatically and gradually improve once immunotherapy is discontinued (8). For some cases with organ dysfunction or severe clinical symptoms, hormonal therapy, like dexamethasone, is highly effective and can rapidly improve and eliminate systemic symptoms (4, 8, 11). Interestingly, such patients with severe adverse events frequently develop superior drug responses and survival outcomes to ICI treatment. It has been demonstrated that ISR is significantly, and positively correlated with the long-term survival of cancer patients (11, 13). Therefore, oncologists support the continual use of immunotherapy following the onset of ISR, due to favorable controllability. A patient with advanced esophageal squamous cell carcinoma, who developed pulmonary sarcoidosis-like reactions induced by sintilimab, was reported to receive the anti-PD-1 inhibitor of sintilimab for one year in a routine, contributing to a manageable safety profile (11). Regarding drug efficacy, the tumor lesions of the patient were kept in a stable condition, showing satisfactory prognostic outcomes (11).

### 2.2 Neoadjuvant immunotherapy for patients with early-stage TNBC

As far as we know, it represents the first case of hepatic sarcoidosis-like reactions induced by neoadjuvant pembrolizumab for patients with early-stage TNBC, which represents a rare adverse event for pembrolizumab. Although ISR have been previously reported in the advanced cancer patients (9–12), it is infrequent in the population of patients with TNBC, especially in the early stage. To see, the case is highly innovative from the perspective of the disease population, as well as the specific type and therapeutic stage of immunotherapy.

Emerging studies have elaborated the clinical benefits of ICIs in TNBC patients, such as the famous KEYNOTE-522 study (2, 14, 15). In the clinical trial, the addition of the PD-1 inhibitor pembrolizumab to chemotherapy as neoadjuvant therapy was proved to be effective for patients with early-stage TNBC, significantly improving pCR rates (short-term efficacy), event-free survival (EFS), and overall survival (OS, long-term efficacy) in the intention-to-treat population (1, 2, 14). Up to now, the survival benefit of pembrolizumab after surgery for those who achieve pCR is controversial. Published data provide some insights into the issue. In the subgroup analyses focusing on patients achieving pCR, EFS and OS were not significant for patients who received pembrolizumab in the post-surgery setting *versus* those who did not, with survival curves almost overlapping (2). It means that the survival benefit of pembrolizumab therapy subsequent to surgery for one year is weak, inferring the limited necessity to continue pembrolizumab in the specific patient population. However, in terms of hazard ratios (HR), pembrolizumab-combined therapy following surgery was suggested to reduce 31% of risks of recurrence or death for patients achieving pCR (HR=0.69, 95% CI = 0.38-1.26)^2^, reflecting profound clinical benefits of pembrolizumab in early-stage TNBC patients with pCR.

Overall, patients with early-stage TNBC are more likely to derive survival benefits when they receive pembrolizumab combined with neoadjuvant chemotherapy compared to those who receive neoadjuvant chemotherapy alone. For the targeted patients with pCR, a great number of factors need to be weighed regarding the subsequent adjuvant immunotherapy lasting one year. They include patients’ own wishes, tolerability and safety, drug accessibility, and economic conditions.

## Data Availability

The original contributions presented in the study are included in the article/supplementary material. Further inquiries can be directed to the corresponding authors.
