# Supplementary material for: Case Report: Hepatic sarcoidosis-like reaction from neoadjuvant pembrolizumab in early-stage triple-negative breast cancer
Source: Front Immunol. 2025 Jun 18;16:1589191. doi: 10.3389/fimmu.2025.1589191 (PMC12213625; doi:10.3389/fimmu.2025.1589191)
Supplement: Supplementary file 1 [file DataSheet1.pdf]

Figure legend

Figure S1 Images of the pre-treatment liver in baseline.

Figure S2 Dynamic changes of the lesion in the left lobe of the liver.

Figure S3 Dynamic changes in the size of the left breast tumors and left axillary lymph nodes during neoadjuvant therapy.

Table legend

Table S1 Overview of ICI induced sarcoidosis-like reaction.

Figures

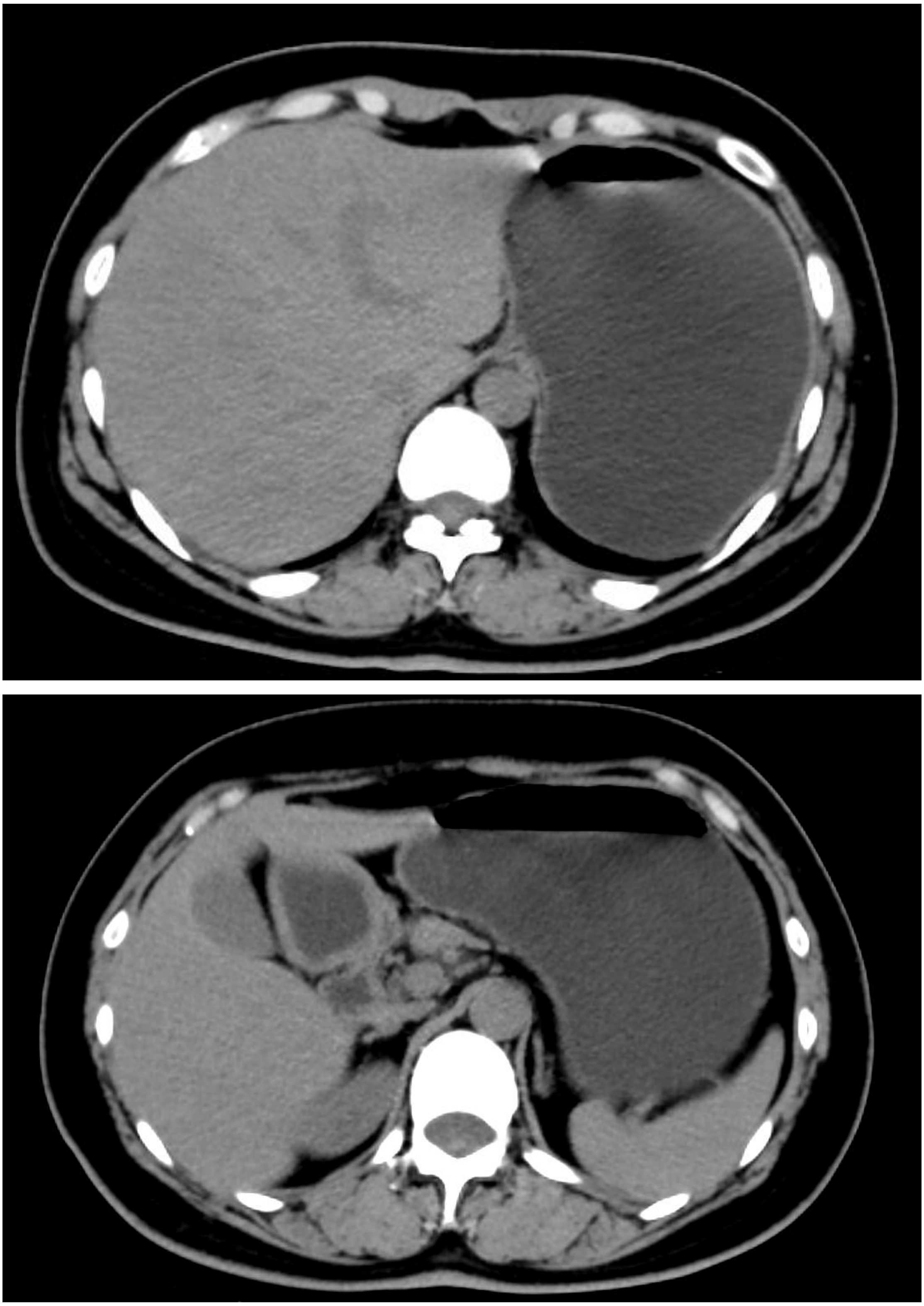

Figure S1 Images of the pre-treatment liver in baseline.

A

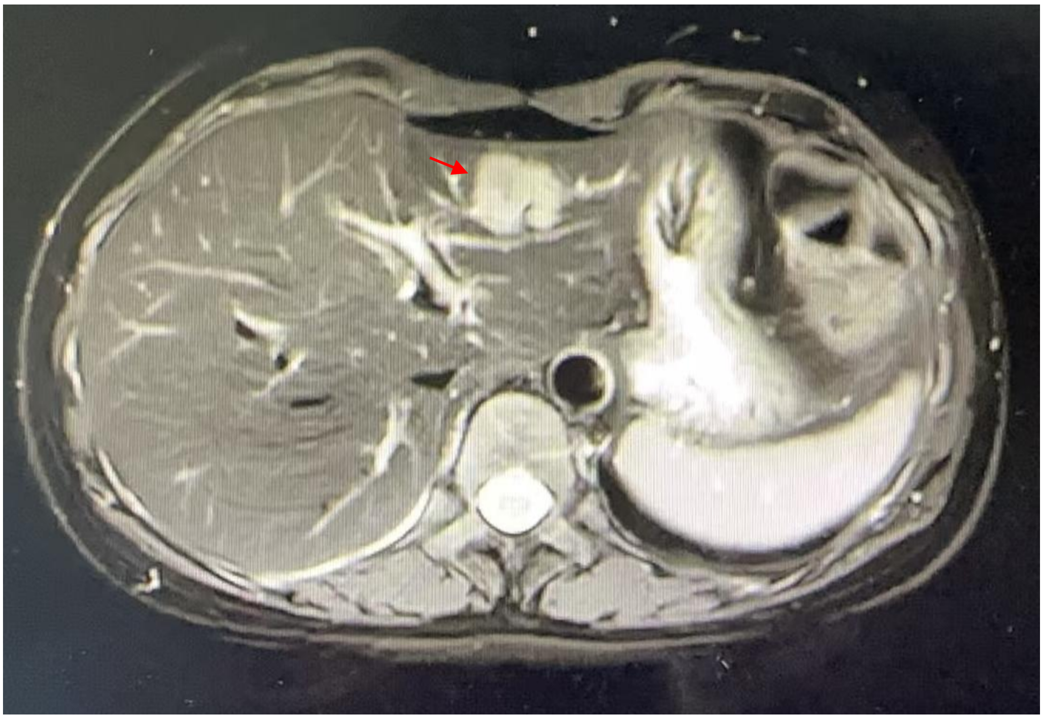

B

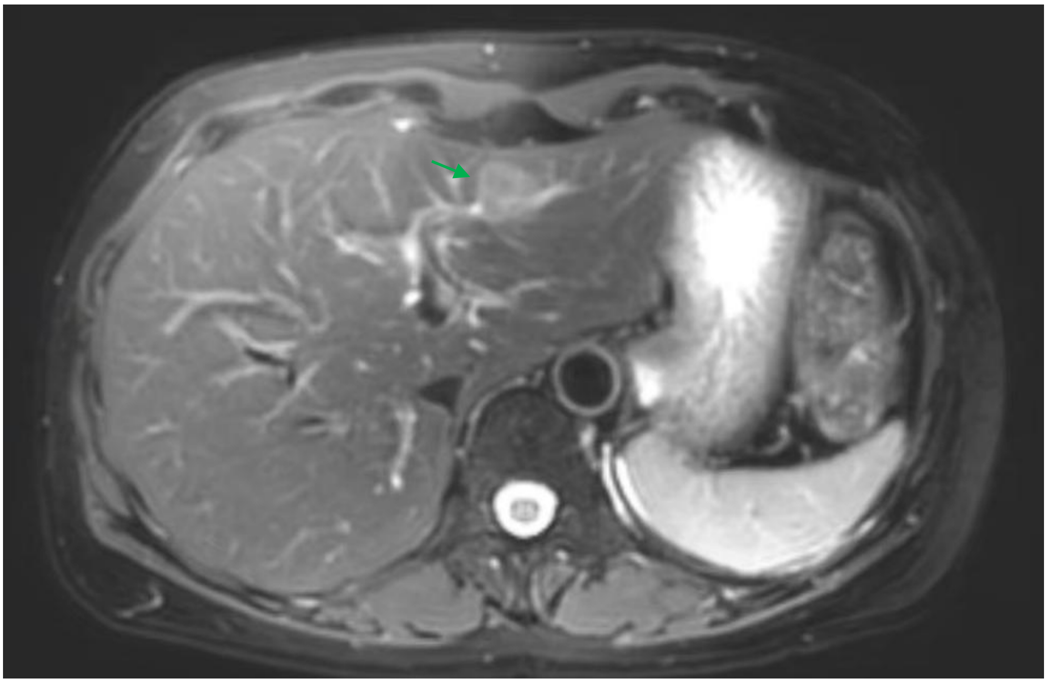

C

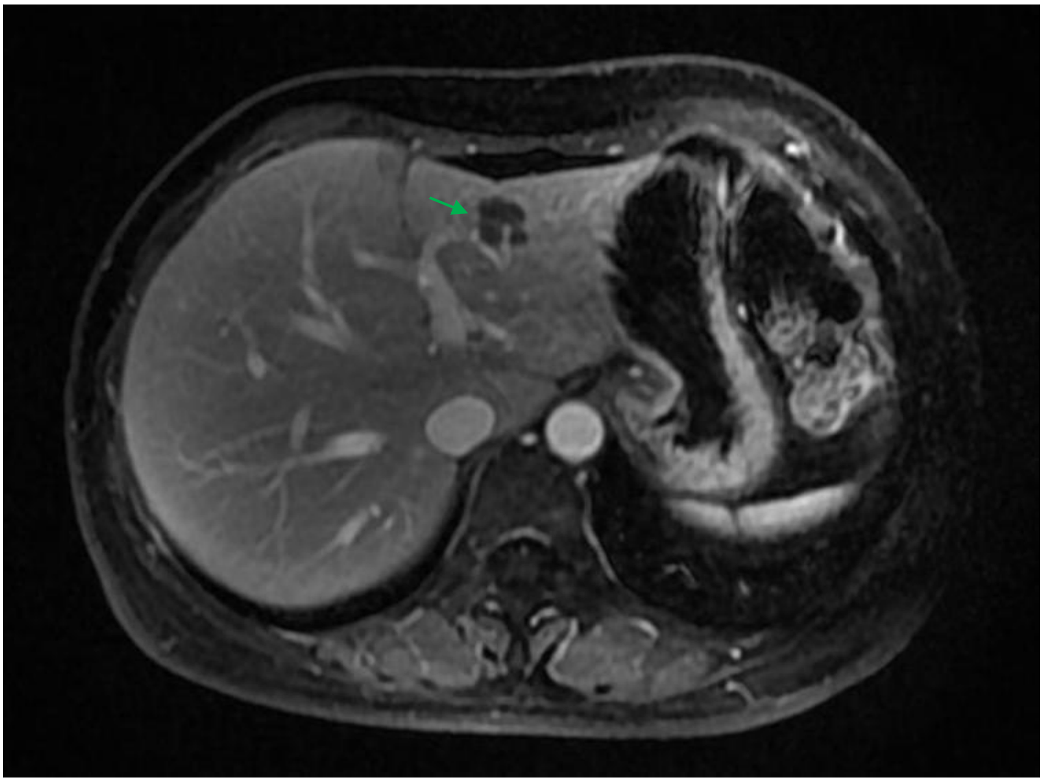

D

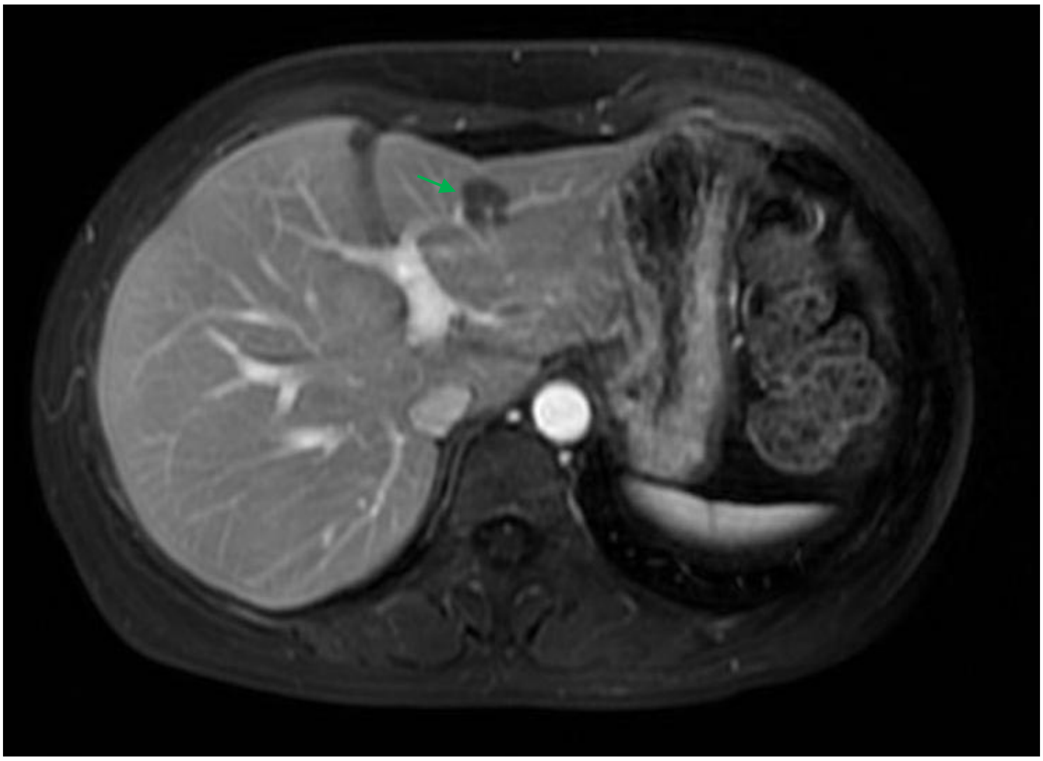

Figure S2. Dynamic changes of the lesion in the left lobe of the liver. (A) The liver lesion in the left lobe with cystic changes (2.4 x 2.4 x 3.0 cm) was identified after six cycles of neoadjuvant therapy with pembrolizumab, which is marked by the red arrow. The size of the liver lesion was reduced after stopping pembrolizumab for several months. The green arrow indicates the smaller lesion. (B)

Mid-October of 2024: 1.7x1.6 cm, (C) Early January of 2025: 1.7x1.6 cm, (D) Late April of 2025: 1.7x1.6 cm.

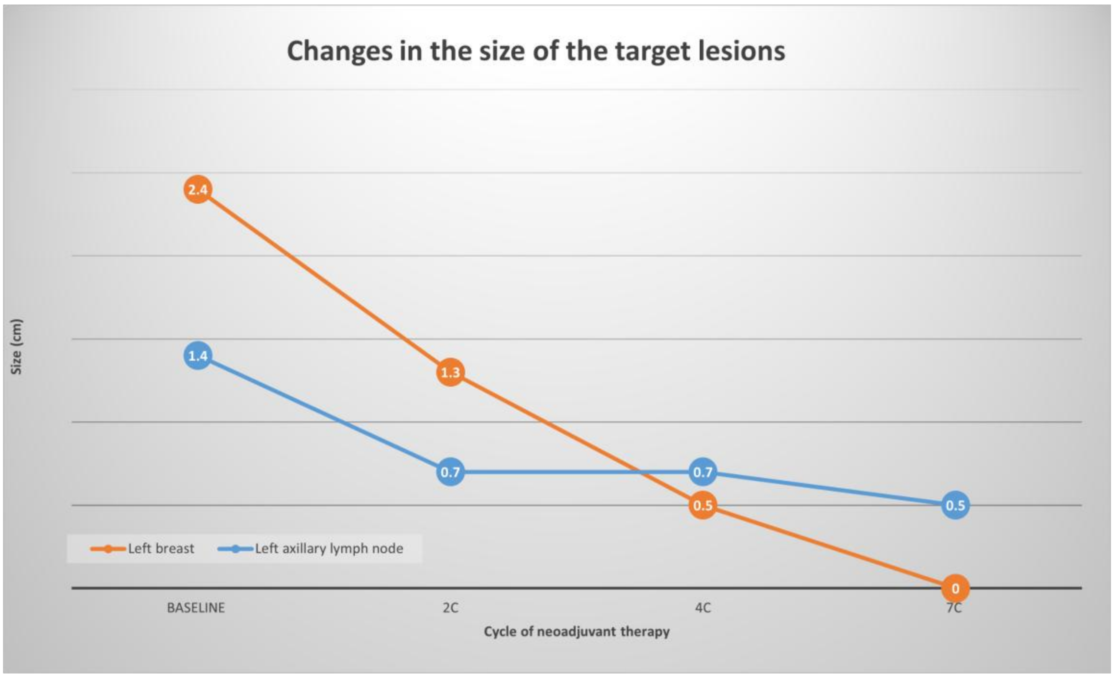

Figure S3. Dynamic changes in the size of the left breast tumors and left axillary lymph nodes during neoadjuvant therapy. Both left breast tumors and left axillary lymph nodes were evaluated to be gradually decreased.

Table S1 Overview of ICI induced sarcoidosis-like reaction

**Cancer types**	**Specific ICIs**	**Reactions**	**Accompanying symptoms**
Melanoma[1,2]	Pembrolizumab	Multiple enlarged lymph nodes	Diabetic ketoacidosis, hypothyroidism
Hepatocellular carcinoma[3]	Nivolumab and ipilimumab	Multiple enlarged lymph nodes, splenomegaly	Fever, uveitis in both eyes
Renal cell carcinoma[4]	Nivolumab and ipilimumab	Enlarged mediastinal lymph nodes	-
Advanced TNBC[5]	Atezolizumab	Enlarged hilar lymph nodes, subcutaneous nodules of extremities	-
Non-small-cell lung carcinoma[6]	Pembrolizumab	Multiple epithelioid cell granulomas in renal interstitium	Renal failure
Esophageal squamous cell carcinoma[7]	Sintilimab	Pulmonary sarcoidosis-like reactions	-

Abbreviations: immune checkpoint inhibitors (ICIs), triple-negative breast cancer (TNBC).

References for Table S1:

1.Li, Y et al. Clinical radiology vol. 78,2 (2023): e131-e136.

2. Cao, Yuanzhen et al. Melanoma research vol. 34,1 (2024): 70-75.

3.Torres-Zurita, Alberto et al. Frontiers in immunology vol. 14 1150128.

4. Rizzo, Mimma et al. Immunotherapy vol. 16,9 (2024): 603-609.

5. Tsunoda, Akira et al. *Case reports in oncological medicine* vol. 2022 2709062.

6. Park, Sang-Don et al. *Medicina (Kaunas, Lithuania)* vol. 59,5 991.

7. Li, Haoqian et al. *Medicine* vol. 102,31 (2023): e34432.
